# Transforming Cardio-Oncology Care Through AI-Driven Large Language Model Systems: A Roadmap for Future Implementation

**DOI:** 10.1016/j.jacadv.2025.102117

**Published:** 2025-08-29

**Authors:** Aryan Agar, Viraj Shah, Harikrishnan Hyma Kunhiraman, Tarek Nahle, Anant Madabhushi, Irbaz Bin Riaz, Momita Bandaru, Tochukwu M. Okwuosa, Nathanael Fillmore, Avirup Guha

**Affiliations:** a Wheeler High School, Cobb County School District, Marietta, Georgia, USA; b Cardio-Oncology Program, Medical College of Georgia at Augusta University, Augusta, Georgia, USA; c Division of Cardiology, Department of Medicine, Medical College of Georgia at Augusta University, Augusta, Georgia, USA; d Wallace H. Coulter Department of Biomedical Engineering, Georgia Institute of Technology and Emory University, Atlanta, Georgia, USA; e Atlanta Veterans Affair Medical Center, Atlanta, Georgia, USA; f Division of Medical Oncology, Department of Medicine, Mayo Clinic, Phoenix, Arizona, USA; g Division of Cardiology, Rush University Medical Center, Chicago, Illinois, USA; h Massachusetts Veterans Epidemiology Research and Information Center, Boston, Massachusetts, USA

**Keywords:** artificial intelligence, cardio-oncology, electronic health records, large language models, retrieval-augmented generation

Artificial intelligence (AI) is poised to transform cardio-oncology by enhancing clinical workflows, improving patient outcomes, and addressing the cardiovascular risks associated with cancer therapies. Large language models (LLMs) have already demonstrated value in tasks such as clinical note summarization, electronic health record (EHR) data extraction, and patient-specific treatment planning. In cardiology, LLMs show performance comparable to established cardiovascular risk scores, while in oncology, LLM tools have achieved real-world accuracy in patient education and clinical trial matching. Despite this promise, most applications remain task-specific and are not yet integrated into daily cardio-oncology workflows.^1^, ^2^, ^3^

Training a foundation LLM from scratch requires massive, heterogeneous data sets and significant computational resources—an approach that remains impractical for most health systems. In contrast, fine-tuning or augmenting an existing LLM using institution-specific data and domain knowledge offers a more feasible and scalable path. To enable broader adoption, LLMs must be fine-tuned with targeted data sets, embedded with expert-derived resources, and validated across diverse populations. We propose a modular, AI-powered assistant—leveraging retrieval-augmented generation (RAG) and institutional customization—to unify triage, cardiotoxicity monitoring, evidence synthesis, and clinical decision support within existing EHR systems.

Augmenting an LLM requires modifying a pretrained model to perform a specific task or providing it with additional information and experience. Several critical steps (Figure 1) are involved in this process especially to fine tune these models in line with the health institution’s needs.

**Figure 1:**
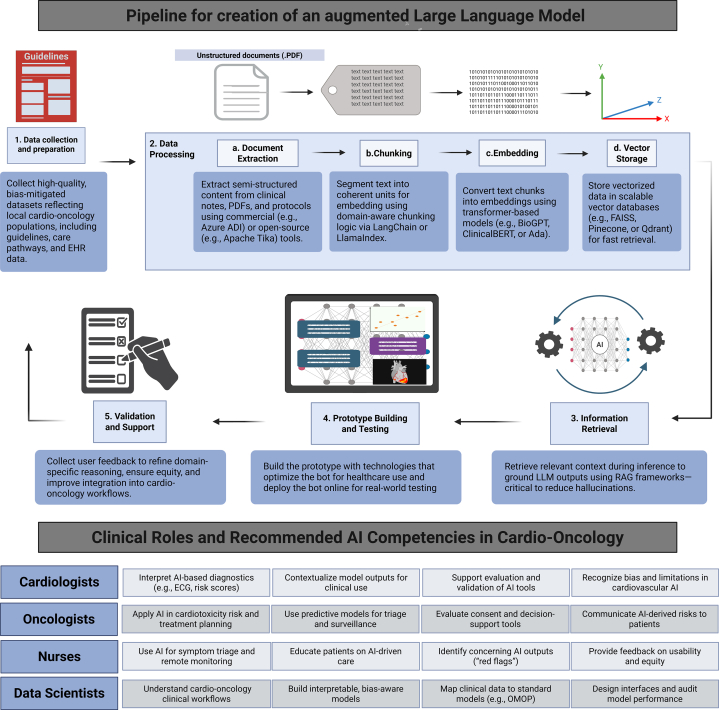
**Pipeline for Creation of an Augmented Large Language Model Framework for augmenting LLMs in Cardio-Oncology: Pipeline and AI Competencies** The top panel illustrates the pipeline for augmenting a LLM using clinical data and institutional knowledge. Key stages include: 1) data collection and preparation; 2) processing and chunking of clinical documents; 3) embedding and vector storage; 4) information retrieval and prototype deployment; and 5) validation and support via clinician feedback loops. The process integrates tools such as LangChain, BioGPT, and ClinicalBERT while emphasizing RAG to minimize hallucinations and enhance contextual accuracy. The bottom panel outlines recommended AI competencies across clinical roles—cardiologists, oncologists, nurses, and data scientists—needed to ensure safe, equitable, and effective integration of AI tools in cardio-oncology care. Created in https://BioRender.com. ADI = Azure Document Intelligence; AI = artificial intelligence; EHR = electronic health record; LLM = large language model; OMOP = Observational Medical Outcomes Partnership; RAG = retrieval-augmented generation.

To fine-tune or ground an LLM for cardio-oncology, a curated, high-quality data set is essential. This data set should accurately reflect the local patient population, contain clinically relevant information including, social determinants of health and be developed with attention to bias mitigation. Assessing and addressing bias involves evaluating model performance across key subgroups (eg, by age, race/ethnicity, sex), analyzing data completeness, and conducting ongoing monitoring for disparities in outputs. In addition to clinical data, the integration of expert-derived resources, such as institutional protocols, cardio-oncology care pathways, and professional society guidelines can further enhance the model’s contextual accuracy and clinical utility.

Once the data set and domain knowledge are integrated, an appropriate LLM must be selected. We evaluated multiple models—including GPT-4, Gemini 1.5, and LLaMA-3-70B—for their suitability in clinical applications. GPT-4 was ultimately selected due to its strong performance on medical benchmarks (e.g., MedQA and PubMedQA), a low hallucination rate (∼2.3%), and large context windows (up to 128k tokens) with relatively fast response times (∼250 ms/token). While open-source models like LLaMA-3-70B offered greater transparency and ease of deployment, they demonstrated higher hallucination rates (∼3.4%) and reduced context capacity (65k tokens). Domain-specific models such as ClinicalBERT and BioGPT, though valuable for classification tasks, were limited by short context lengths (512-1,000 tokens) and higher hallucination rates (>6.5%), rendering them less suitable for real-time generative tasks in cardio-oncology.

To further strengthen contextual performance, we employ RAG, a framework that enables the model to retrieve up-to-date medical information from curated external sources at inference time, rather than relying solely on pretraining.^4^^,^^5^ This approach enhances domain specificity, transparency, and adaptability.

The prototype AI-driven assistant also incorporates several key technologies to support real-world use:

• Azure Document Intelligence (ADI): Extracts and structures information from medical literature and clinical notes.^4^ ADI was selected for its Health Insurance Portability and Accountability Act (HIPAA) compliant infrastructure, support for health care—specific templates, and prebuilt AI models optimized for medical content parsing. Unlike general-purpose Optical Character Recognition tools, ADI can recognize tabular formats, detect handwritten physician notes, and automatically categorize clinical content into Fast Healthcare Interoperability Resources (FHIR)-compatible fields. This makes it particularly suited for preprocessing heterogeneous EHR data in cardio-oncology workflows.
• LangChain: Enables integration of clinical guidelines and patient-specific EHR data across platforms.^5^ It is framework that facilitates orchestration of LLM workflows by chaining together multiple data sources and logic steps. LangChain was selected for its ability to support multimodal input pipelines, custom retrieval agents, and integration with EHR backend through modular application programming interface (API) connectors.
• Streamlit: Provides a user-friendly interface for health care professionals to interact with the AI tool.^5^ Streamlit was chosen for its developer efficiency, open source compatibility with Python-based clinical AI tools, and low infrastructure overhead. Its ability to deploy real-time, browser-based dashboards makes it ideal for clinician-facing interfaces.

This assistant, hosted on Streamlit Cloud, will parse documents, answer cardio-oncology queries, and offer real-time decision support. Its reliability will improve over time through user feedback, which informs local fine-tuning and ensures alignment with institutional workflows.

Effective AI implementation in cardio-oncology requires a structured, multidimensional approach. First, AI models must be integrated into existing EHR systems to enable seamless clinical utility. Model training should be drawn from diverse, high-quality data sets—such as imaging, electrocardiograms, laboratory values, and clinical notes—to enhance predictive accuracy and relevance. Incorporating RAG allows real-time access to clinical guidelines and patient-specific data, further improving contextual precision.

Second, AI systems should serve as decision-support tools that augment, not replace, clinician judgment (Figure 1). Even when trained on clinically meaningful and bias-mitigated data, models may still produce inaccuracies or hallucinations. Transparent validation protocols are essential to detect and mitigate these errors, safeguarding clinical decision-making and patient safety.

Third, accessibility and equity must guide AI deployment. The uneven distribution of cardio-oncology expertise, especially in rural and socioeconomically disadvantaged settings, amplifies the need for virtual consultation platforms and remote monitoring systems. However, digital access barriers remain significant: 43% of U.S. adults with household incomes under $30,000 lack home broadband, and nearly one-quarter do not own a smartphone. These disparities are further exacerbated by lower educational attainment and rurality.^6^ To address this, AI systems should support offline-first capabilities, be optimized for mobile and low-bandwidth environments, and utilize federated learning—enabling decentralized model training without centralized data transfer.

Finally, real-world implementation must confront persistent data challenges. Clinical data sets often lack longitudinal continuity, contain missing or incomplete data, and are biased toward certain institutions or populations. Rather than viewing these limitations solely as obstacles, institutions must develop strategies to adapt local data inconsistencies for contextual advantage. EHR heterogeneity including variation in data formats, terminologies, and documentation practices further undermines interoperability and limits generalizability. Common data models, such as Observational Medical Outcomes Partnership, provide a pathway toward standardization, though widespread adoption remains uneven. As underscored in the 2024 American Heart Association Scientific Statement on AI in Cardiovascular Care, achieving equitable and clinically meaningful model performance requires deliberate inclusion of underrepresented populations, harmonized data curation, and rigorous external validation across diverse health care settings.^7^

AI also holds significant promise for transforming clinical trials in cardio-oncology. LLMs can enable adaptive enrichment by analyzing EHR and biomarker data to identify patient subgroups most likely to benefit from investigational cardioprotective therapies. AI-driven recruitment tools—such as OncoLLM—have demonstrated physician-level accuracy in matching patients to clinical trials, while platforms like TrialGPT have streamlined eligibility screening and reduced clinician workload. Generative AI-powered eConsent chatbots further enhance the trial experience by presenting information in accessible, lay-friendly language and responding to participant questions in real time, thereby improving comprehension and enrollment rates. Collectively, these tools have the potential to mitigate long-standing disparities in trial access and understanding. Notably, the U.S. Food and Drug Administration’s 2024 workshop on generative AI underscored the importance of transparency, adaptive design, and federated learning in future clinical development frameworks—signaling a growing regulatory readiness to support these innovations.^2^^,^^3^^,^^8^^,^^9^

Currently, in 2025, the U.S. Food and Drug Administration has drafted a guide on how it is considering regulating AI-based medical devices, which proposes total product lifecycle management and ongoing monitoring of AI-driven medical tools.^10^ Alongside this, the Office for Civil Rights has increasingly been targeting dubious AI usage in patient care by enforcing an updated HIPAA Security Rule. This pushes for audits for the tracking technologies used in AI workflows. The integration of AI into any medical field raises questions about trust, transparency, and bias. Clinicians and patients have to be able to understand how these AI-generated answers are derived, especially in high-stress environments like the medical field. Bias auditing must be embedded into model development.

AI-powered tools, especially LLMs, have the potential to transform cardio-oncology by improving decision-making, expanding access, and supporting clinical teams. Realizing this promise requires: 1) domain-specific fine-tuning and integration into EHR workflows; 2) equipping multidisciplinary teams with clear, role-based competencies (Figure 1); and 3) ensuring equity through bias-aware data sets and inclusive deployment strategies. With thoughtful implementation, AI can close care gaps and elevate outcomes for patients facing both cancer and cardiovascular disease.

## Funding support and author disclosures

Dr Guha is supported by 10.13039/100000968American Heart Association-Strategically Focused Research Network Grant in Disparities in Cardio-Oncology (#847740, #863620) and Department of Defense Prostate Cancer Research Program's Physician Research Award (#HT94252310158). Dr Okwuosa is on the editorial board for the Journal of the American Heart Association. Dr Fillmore has received institutional research funding from Merck and Bayer. Dr Madabhushi is involved in 2 different R01 grants with Inspirata Inc; has been licensed to Picture Health and Elucid Bioimaging; has received consulting fees from Takeda Inc; has served as a member of the Frederick National Laboratory Advisory Committee; has served as a Advisory board of Picture Health and SimBioSys; owns stock or stock options for Picture Health, Elucid Bioimaging, and Inspirata Inc; and has sponsored research agreements with AstraZeneca and Bristol Myers Squibb. All other authors have reported that they have no relationships relevant to the contents of this paper to disclose.
